# Anti-interleukin-6 receptor antibody reduces neuropathic pain following spinal cord injury in mice

**DOI:** 10.3892/etm.2013.1296

**Published:** 2013-09-13

**Authors:** TOMOTOSHI MURAKAMI, TSUKASA KANCHIKU, HIDENORI SUZUKI, YASUAKI IMAJO, YUICHIRO YOSHIDA, HIROSHI NOMURA, DAN CUI, TOSHIZO ISHIKAWA, EIJI IKEDA, TOSHIHIKO TAGUCHI

**Affiliations:** 1 Department of Orthopedic Surgery, Yamaguchi University Graduate School of Medicine, Ube, Yamaguchi 755-8505;; 2 Department of Orthopedic Surgery, Hiroshima Red Cross Hospital, Hiroshima, Hiroshima 730-0052;; 3 Departments of Pathology, Yamaguchi University Graduate School of Medicine, Ube, Yamaguchi 755-8505, Japan; 4 Neurosciences, Yamaguchi University Graduate School of Medicine, Ube, Yamaguchi 755-8505, Japan

**Keywords:** spinal cord injury, inflammation, interleukin-6, hyperalgesia, allodynia

## Abstract

The present study reports the beneficial effects of an anti-mouse interleukin-6 (IL-6) receptor antibody (MR16-1) on neuropathic pain in mice with spinal cord injury (SCI). Following laminectomy, contusion SCI models were produced using an Infinite Horizon (IH)-impactor. MR16-1 was continuously injected for 14 days using Alzet osmotic pumps. A mouse IL-6 ELISA kit was then used to analyze IL-6 levels in the spinal cord tissue between 12 and 72 h after injury. Motor and sensory functions were evaluated each week using the Basso Mouse Scale (BMS), plantar von Frey and thermal threshold tests. Histological examinations were performed 42 days after SCI. Between 24 and 72 h after SCI, the expression levels of IL-6 were significantly decreased in the MR16-1 treated group. Six weeks after surgery, the BMS score of the MR16-1-treated group indicated significant recovery of neurological functions. MR16-1-treated mice in the SCI group exhibited lower paw withdrawal thresholds in the plantar von Frey and thermal tests, which were used to evaluate allodynia. MR16-1 treatment significantly increased the area of Luxol fast blue-stained tissue, representing spared myelin sheaths. These results indicate that the continuous inhibition of IL-6 signaling by MR16-1 between the early and sub-acute phases following SCI leads to neurological recovery and the suppression of hyperalgesia and allodynia. Overall, our data suggest that the inhibition of severe inflammation may be a promising neuroprotective approach to limit secondary injury following SCI and that an anti-IL-6 receptor antibody may have clinical potential for the treatment of SCI.

## Introduction

Over two-thirds of individuals with spinal cord injury (SCI) experience the effects of neuropathic pain in their daily lives. Neuropathic pain is resistant to general analgesic treatment and is a long-term issue for SCI patients. SCI causes severe motor and sensory dysfunction, while neuroinflammation is an important secondary event in the injury cascade. The development of strategies to minimize this auto-destructive injury is one of the main aims in the field of SCI research. A number of studies have demonstrated remarkable protection and functional recovery using anti-inflammatory reagents in SCI models (1–7). However, to date, there have been no studies concerning the use of anti-inflammatory reagents to reduce neuropathic pain following SCI. The interleukin-6 (IL-6) cytokine is important in mediating pro-inflammatory damage after SCI (8–10). Activation of the Janus kinase and signal transducer and activator of transcription 3 (JAK-STAT3) signaling pathway by IL-6 is an important mechanism for transducing signals from the cell surface and is strongly linked to immune/inflammatory reactions (11,12). Early activation of this pathway occurs most often in spinal microglia and contributes to the development of neuropathic pain (13–15). Attenuation of IL-6 activity is therefore an attractive therapeutic strategy for reducing the neurological deficits associated with SCI. The present study reports a significant reduction of neuropathic pain in mice with SCI following the administration of anti-mouse IL-6 receptor antibody (MR16-1).

## Materials and methods

### Experimental procedures

All experiments were approved by the Ethics Committee for Animal Studies at Yamaguchi University (Ube, Japan) and were carried out in accordance with the Guidelines for Proper Conduct of Animal Experiments, Science Council of Japan (June 1, 2006).

### MR16-1

The rat anti-mouse IL-6 receptor monoclonal antibody (MR16-1), a gift from Chugai Pharmaceuticals Co. Ltd., (Tokyo, Japan), was prepared as described previously (16). An isotype of this antibody is IgG1. MR16-1 has been shown to bind to the soluble mouse IL-6 receptor and suppress IL-6-induced cellular responses in a dose-dependent manner. Other basic characterizations of this antibody have been described in previously published reports (8).

### Animals and surgery

Sixty adult female C57BL/6J mice (10 weeks old) were obtained from Japan SLC, Inc. (Shizuoka, Japan) and assigned to the following groups: The MR16-1 group, comprising MR16-1-treated mice (n=25); the control group, comprising untreated SCI mice (n=25); and the sham group, comprising mice subjected to laminectomy but with normal spinal cords (n=10). Mice were anesthetized with an intraperitoneal injection of ketamine (100 mg/kg) and xylazine (10 mg/kg). Laminectomy was performed at the level of the 10th thoracic vertebra under a surgical microscope. A contusion SCI model was produced using an Infinite Horizon (IH)-impactor (PSI Inc., Lexington, KY, USA) with an impact force of 60 kdyn (17). Immediately after injury, MR16-1 was continuously injected for 1–14 days (150*μ*g/day) using Alzet osmotic pumps (DURECT Corporation., Cupertino, CA, USA).

### ELISA analysis of interleukin-6 concentration

Spinal cord tissue (5 mm in length) at the lesion epicenter was dissected in each group (5 animals per group) at 12, 24 and 72 h after injury. Spinal cord tissue samples to be used for ELISA analysis were homogenized in radio-immunoprecipitation assay (RIPA) lysis buffer (500 *μ*l; Santa Cruz Biotechnology, Inc., Santa Cruz, CA, USA) and the IL-6 concentration was measured using a mouse IL-6 ELISA kit (Invitrogen Life Technologies, Carlsbad, CA, USA) according to the manufacturer’s instructions. The IL-6 levels were expressed in pg/mg (18).

### Assessment of motor function recovery

The Basso Mouse Scale (BMS) is a validated scale used to monitor the progress of hind-limb functional recovery following SCI. The scale ranges from 0 (no ankle movement) to 9 (complete functional recovery) points (19). BMS scores were recorded at 3, 7, 14, 21, 28 and 42 days following SCI by two independent examiners who were blind to the experimental conditions. Hind-limb motion was used to assess coordinated movement and stepping. When differences in the BMS score between the right and left hind limbs were observed, the average of the two scores was used.

### Assessment of sensory function recovery and allodynia

Sensory tests were performed 21 and 42 days after SCI. All behavioral tests were conducted by an experienced investigator who was blind to the type of intervention. Each hind paw was tested three times. Paw withdrawal latencies to heat were measured according to the Hargreaves’ method (20) by applying a standard Plantar test (Ugo Basile, Comerio, Italy). The animals were placed on a glass surface and a radiant heat source was positioned under one hind paw. The latency to paw withdrawal was recorded automatically. Paw withdrawal thresholds to tactile stimulation were measured according to the von Frey test using a standard Dynamic Plantar Aesthesiometer (Ugo Basile). The animals were placed in plexiglass cages on a wire mesh. The plantar surface of the hind paw was probed with a von Frey monofilament (21) and the force required for paw withdrawal was recorded automatically. The von Frey filament and thermal threshold tests were used to measure mechanical allodynia and thermal hyperalgesia, respectively.

### Histological analysis

Spinal cords were harvested 42 days after surgery. Mice were anesthetized with an intraperitoneal injection of ketamine (100 mg/kg) and xylazine (10 mg/kg) and then perfused with 4% paraformaldehyde. A 1-cm length of spinal cord that included the lesion center was removed and frozen for sectioning. The tissue was sectioned axially in 10-*μ*m-thick sections. Transverse sections from the injury epicenter were also stained for myelin using Luxol fast blue (LFB). LFB-positive areas in which the density significantly exceeded the threshold of each background were calculated as the percentage cross-sectional area of residual tissue. Tissue sections were analyzed using the Cavalieri Probe (Stereo Investigator 64-bit software; MBF Bioscience, Williston, VT, USA) (22).

### Electrophysiological evaluation

Sensory evoked potentials (SEPs) were recorded from the MR16-1 (n=10), control (n=10) and sham (n=5) groups 42 days after SCI. SEPs following sciatic nerve electrical train stimulation were recorded from the sensory cortex of the brain in mice anesthetized with ketamine. A ground electrode was inserted subcutaneously between the stimulating and recording electrodes and a constant current stimulus (S) of 0.1 msec duration and 2.0 mA intensity was applied at a rate of 5.7 Hz to the hind paw. At a band-width of 10–3,000 Hz, a total of 200 traces were averaged and replicated (23). SEP peak latency and amplitude were measured from the start of S to the peak of the first positive peak (P1).

### Statistical analysis

All data in this study are expressed as the mean ± SEM. ELISA data and electrophysiological latency data were analyzed using a one-way ANOVA. The BMS and histological data were analyzed using a two-way ANOVA for repeated measures. Significant ANOVA results were followed by post-hoc Bonferroni analysis. Sensory variables showed normal distribution in the Kolmogorow-Smirnow test. P<0.05 was considered to indicate a statistically significant difference.

## Results

### ELISA data

**Figure 1.**
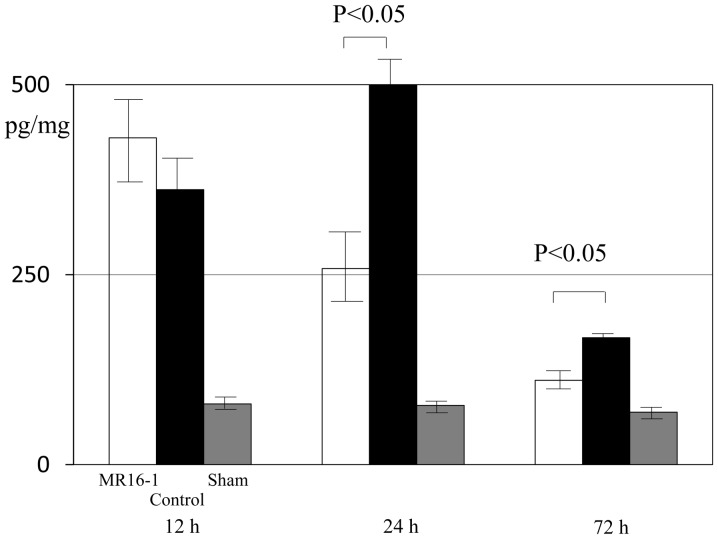
ELISA data. Time course for the expression of IL-6 in spinal cord tissue following SCI. The graph shows IL-6 expression in the normal spinal cords of mice from the sham group. No significant differences in the expression of IL-6 were identified between the MR16-1 and control groups 12 h after SCI. Between 24 and 72 h after SCI, the expression of IL-6 in injured spinal cord tissue was significantly lower in the MR16-1 group than in the control group. SCI, spinal cord injury; IL-6, interleukin-6; MR16-1, anti-mouse IL-6 receptor antibody.

At 12 h after SCI, the expression levels of IL-6 were 430.6±66.8, 362.1±42.3 and 80.0±12.0 pg/mg in the MR16-1, control and sham groups, respectively. No significant differences in the expression levels of IL-6 in the spinal cord were identified between the MR16-1 and control groups 12 h after SCI. At 24–72 h after SCI, the expression of IL-6 in injured spinal cord tissue was significantly lower in the MR16-1 group than in the control group (Fig. 1).

At 24 h following SCI, the expression levels of IL-6 were 258.0±44.7, 503.3±24.0 and 78.0±12.0 pg/mg in the MR16-1, control and sham groups, respectively. At 72 h after SCI, the expression levels of IL-6 were 111.2±6.9, 166.4±5.0 and 77.0±11.5 pg/mg in the MR16-1, control and sham groups, respectively. Between 72 h and 2 weeks following SCI, no significant differences were identified in the expression levels of IL-6 in the spinal cord among the three groups.

### Motor function

**Figure 2.**
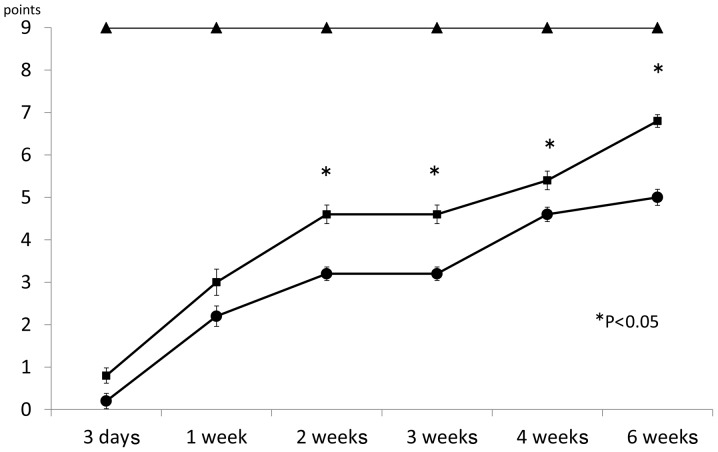
Time course of BMS score following SCI. Between 2 and 6 weeks after SCI, the MR16-1 group exhibited significantly improved recovery compared with the control group. BMS, Basso Mouse Scale; SCI, spinal cord injury; MR16-1. anti-mouse interleukin-6 receptor antibody; ■, BMS scores for the MR16-1 group; ●, BMS scores for the control group; ▲, BMS scores for the sham group. ^*^P<0.05, MR16-1 group vs. control group.

Mice injected with MR16-1 showed continuous recovery of motor function. Three days after surgery, the BMS score was 0.8±0.18 for the MR16-1 group, 0.2±0.18 for the control group and 9.0±0.00 for the sham group (Fig. 2). One week after surgery, the BMS score was 3.0±0.31 for the MR16-1 group, 2.2±0.24 for the control group and 9.0±0.00 for the sham group. Two weeks after surgery, the BMS score was 4.6±0.22 for the MR16-1 group, 3.2±0.16 for the control group and 9.0±0.00 for the sham group. Three weeks after surgery, the BMS score was 4.6±0.22 for the MR16-1 group, 3.2±0.16 for the control group and 9.0±0.00 for the sham group. Four weeks after surgery, the BMS score was 5.4±0.22 for the MR16-1 group, 4.6±0.17 for the control group and 9.0±0.00 for the sham group. Six weeks after surgery, the BMS score was 6.8±0.15 for the MR16-1 group, 5.0±0.19 for the control group and 9.0±0.00 for the sham group. Between 2 and 6 week after SCI, the BMS scores between the MR16-1 and sham groups were significantly different.

The BMS scores for the MR16-1 and control groups indicated gradual recovery one week after SCI. However, between 2 and 6 weeks following MR16-1 treatment, mice in the MR16-1 group showed a marked recovery compared with those in the control group (Fig. 2).

### Sensory functional recovery and prevention of allodynia

**Figure 3.**
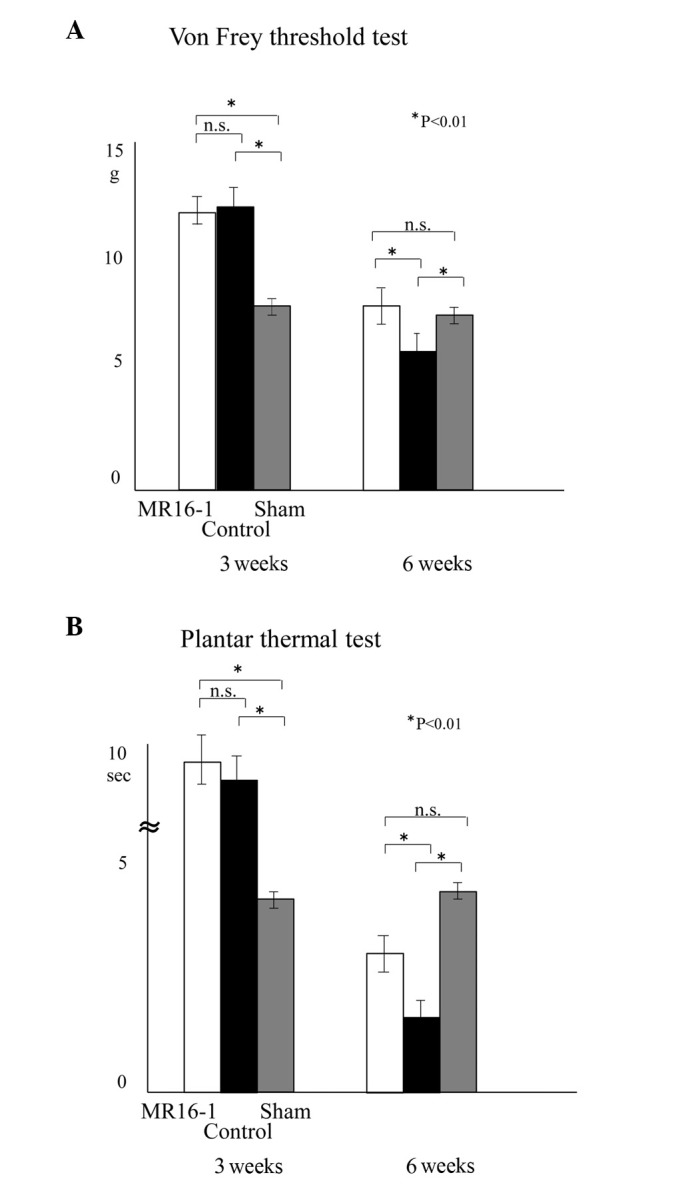
Results of (A) von Frey threshold and (B) plantar thermal tests of sensory functions and allodynia. Three weeks after SCI, the majority of mice were under hypesthesia, thus allodynia could not be evaluated. Six weeks after SCI, hyperalgesia was significantly suppressed in MR16-1 group mice compared with the control group. The data for control group represent the natural course for sensory recovery in SCI mice following a 60-kdyn impact, while the data for the sham group represent normal sensory function without neurological deficit. SCI, spinal cord injury; MR16-1. anti-mouse interleukin-6 receptor antibody; n.s., not significant.

The mice that were continuously infused with MR16-1 showed recovery of sensory function (Fig. 3). The sensory scores of the mice in the von Frey and thermal tests were as follows: in the MR16-1 group 3 weeks after SCI, 11.7±0.64 g and 9.8±0.86 sec, respectively, and at 6 weeks, 7.0±0.84 g and 2.9±0.37 sec, respectively; in the control group 3 weeks after SCI, 11.9±0.69 g and 9.2±0.82 sec, respectively and at 6 weeks, 5.3±1.1 g and 1.7±0.27 sec, respectively; in the sham group 3 weeks after SCI, 6.9±0.26 g and 4.2±0.23 sec, respectively, and at 6 weeks, 6.4±0.24 g and 4.5±0.21 sec, respectively.

At 3 weeks after SCI, the majority of mice were in the recovery phase for sensory function. It was not possible to evaluate the occurrence of allodynia since the majority of mice were under hypesthesia. Six weeks after SCI, hyperalgesia was significantly suppressed in the MR16-1 group compared with the control group. The data for the control group demonstrate the natural course of sensory recovery in SCI, while the data for the sham group demonstrate normal sensory evaluation without neurological deficit.

### LFB stain

**Figure 4.**
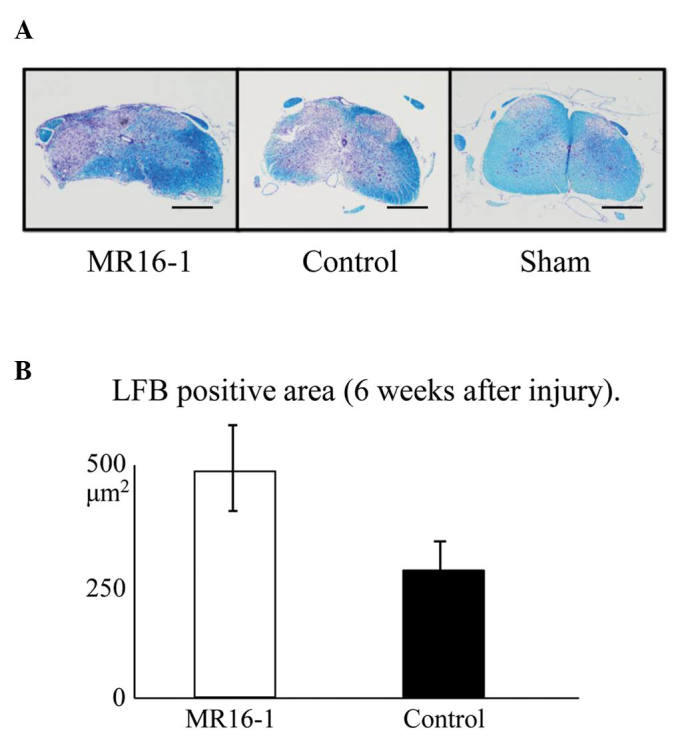
(A) LFB-stained spinal cord at the center of the SCI (scale bar, 50 *μ*m). The LFB-positive area was significantly greater in the MR16-1 group. (B) The area of preserved myelin sheath was significantly higher in the MR16-1 group than in the control group. SCI, spinal cord injury; LFB, luxol fast blue; MR16-1, anti-mouse interleukin-6 receptor antibody.

The area of Luxol fast blue-stained tissue, representing spared myelin sheath, was significantly increased as a result of treatment with MR16-1 (Fig. 4A).

The LFB-positive area at the center of the axial section of the SCI was 482±22 *μ*m^2^ in the MR16-1 group and 263±43 *μ*m^2^ in the control group (Fig. 4B), demonstrating significant preservation in the MR16-1 group.

### SEPs

SEP recordings were performed 6 weeks after SCI in order to provide electrophysiological evidence for the recovery of sensory function. The latency of the first wave was 7.4±1.1 msec in the MR16-1 group, 7.7±0.9 msec in the control group and 7.1±0.5 msec in the sham group (no significant differences were identified). However, the amplitude of the first wave was 9.1±1.9 msec in the MR16-1 group, 4.6±0.8 msec in the control group and 14.1±0.7 msec in the sham group. The amplitude for the MR16-1 group was significantly lower than that of the sham group, but significantly higher than that of the control group. MR16-1-treated SCI mice exhibited suppressed allodynia and increased sensory function, as determined by electrophysiological measurements.

## Discussion

Chronic pain and allodynia following SCI represent a therapeutic challenge. To the best of our knowledge, the present study is the first report demonstrating that MR16-1 suppresses allodynia and hyperalgesia in mice with SCI. Mice treated with MR16-1 exhibited only moderate hyperalgesia of the lower limbs.

A previous study reported that the administration of IL-6 cytokine at lesion sites one day after injury increased the recruitment and the peak of macrophage, neutrophil and microglial cell activity at the lesion sites to a greater extent than when administered 4 days after injury (24). In the present study, the concentration of IL-6 at the injury site between 24 and 72 h after SCI was significantly reduced in mice treated with MR16-1. These mice also exhibited improved locomotor BMS scores from 14 days after SCI compared with untreated mice. This result suggests that MR16-1 conferred a neuroprotective effect in SCI mice. This is in agreement with a previous study in which MR16-1 treatment for SCI decreased connective tissue formation due to astrogliosis (25). The suppression of gliosis leads to protection of the myelin sheath in the injured spinal cord (25). In the present study, an increase in myelin preservation was also observed, as revealed by LFB staining at 42 days post-injury. Furthermore, SEPs confirmed that MR16-1 treatment for SCI led to electrophysiological sensory recovery. In this study, cord dysfunction at the thoracic level was revealed by recording hind-limb SEPs following SCI. Potentials evoked in hind-limbs appeared as well-characterized peaks, and provide a sensitive and quantitative measure to detect pathological changes following SCI. This is in accordance with a number of clinical studies reporting that pathological changes in SEPs are a sensitive method for the detection of the extent of cord injury (23). Although this method primarily represents the function of the somatosensory pathways, SEPs have been used extensively in neurophysiological assessments of spinal cord integrity (23,26).

IL-6 is one of the major proinflammatory cytokines that triggers secondary injury in the pathophysiology of SCI. It is known to promote the activation and infiltration of macrophages and microglia, the major inflammatory cells observed in SCI (9). When IL-6 is released, it binds to the membrane-bound IL-6 receptor (IL-6R) to form an IL-6/IL-6R complex that associates with the signal transducer, gp130, transmitting a signal into cells (16). In addition, the gp130-JAK/STAT pathway promotes the differentiation of astrocytes. These cells produce a chondroitin sulfate proteoglycan (CSPG) that forms a glial scar. Therefore, the overexpression of IL-6 enhances inflammation and tissue injury (25). By contrast, previous studies using gene-knockout animals have revealed that the excessive inhibition of IL-6 signaling is detrimental to functional recovery, since it inhibits axonal regeneration or causes failed gliosis, which implies that IL-6 may also have a beneficial function in spinal cord repair (9,25). Based on these findings and the theory that a blockade of IL-6 signaling may reduce the extent of post-SCI inflammatory damage, previous researchers have administered MR16-1 following SCI and demonstrated reduced inflammation, decreased astrogliosis and enhanced tissue sparing, leading to improved functional recovery.

In conclusion, the present results suggest that continuous blockade of IL-6 signaling following SCI reduces damaging inflammatory activity, thus promoting functional and sensory recovery. A humanized antibody against human IL-6 receptor (MRA; tocilizumab) is already in clinical use for the treatment of rheumatoid arthritis. This drug may also represent a novel option for the treatment of human SCI.
